# Why the renin–angiotensin–aldosterone system (RAAS) in critically ill patients can no longer be ignored

**DOI:** 10.1186/s13054-021-03816-x

**Published:** 2021-11-14

**Authors:** Alexander Zarbock, Lakhmir Chawla, Rinaldo Bellomo

**Affiliations:** 1 Department of Anaesthesiology, Intensive Care and Pain Medicine, University Hospital Münster, Albert-Schweitzer-Campus 1, Geb. A1, 48149 Munster, Germany; 2 Veterans Affairs Medical Center, San Diego, CA USA; 3 Department of Critical Care, The University of Melbourne, Melbourne, Australia; 4 Department of Intensive Care, Royal Melbourne Hospital, Parkville, VIC Australia; 5 Department of Intensive Care, Austin Health, Heidelberg, Australia; 6 Australian and New Zealand Intensive Care Research Centre, School of Public Health and Preventive Medicine, Monash University, Melbourne, Australia

## Background

Vasodilatory shock is a common form of shock, characterized by preserved or increased cardiac output and peripheral vasodilation. Inflammatory syndromes or states, such as sepsis and major surgery, are common causes of vasodilatory shock. Acute kidney injury (AKI) is a common complication of a vasodilatory shock and is associated with increased mortality [1]. Sepsis is the most common cause of AKI [2]. The pathophysiology of vasoplegia is complex and not fully understood, but different hormonal systems are involved in the regulation of systemic vascular resistance, including the renin–angiotensin–aldosterone system (RAAS).

The RAAS contributes to the control of blood pressure, fluid homeostasis, electrolyte balance, and glomerular filtration rate [3]. Renin cleaves angiotensinogen to angiotensin (AT)-I and this precursor is then cleaved to produce AT-II by the angiotensin converting enzyme (ACE). High AT-II levels produce vasoconstriction, stimulate the adrenal gland to release aldosterone and, in turn, inhibit renin generation by biofeedback. Conversely, if AT-II-generation is impaired renin-levels will rise in an attempt to generate more AT-II precursor.

In a recent study, Flannery and colleagues [4] demonstrated that elevated renin-levels in critically ill patients are associated with worse outcomes. In a multicenter study, the authors included critically ill patients with (at least stage 2) or without AKI. The primary outcome was the composite endpoint of major adverse kidney events (MAKE) at hospital discharge, consisting of mortality, kidney replacement therapy, or reduced estimated glomerular filtration rate to ≤ 75% of baseline. The MAKE rate was twice as high in patients in the top renin-tertile compared with the bottom renin-tertile, mortality was three fold greater and the use of kidney replacement therapy was four-fold greater. On multivariable logistic regression analysis, renin-levels remained significantly associated with MAKE. However, the authors were unable to adjusted renin-levels for the recent intake of ACE inhibitors or ARBs and did not report on renin-levels according to the presence of vasodilatory shock. Nonetheless, patients in the upper tertile of renin-levels received more vasopressor drugs at baseline (*P* < 0.001).

These findings are in line with previous work also showing that elevated renin-levels are associated with an increased mortality [5]. In this post-hoc analysis, the authors demonstrated that renin-levels are commonly elevated in patients with catecholamine-resistant vasodilatory shock. Moreover, they found that renin-levels correlated with the AT-I/AT-II ratio, which was also increased [5]. The underlying cause of this finding might be related to ACE-dysfunction or increased AT-II degradation by neutral endopeptidase. The inflammatory response, which accompanies critical illness, however, causes a reduced ACE-activity. This reduction may subsequently lead to decreased conversion of AT-I to AT-II [5] which will likely lead to persistent hypotension and high renin-levels. Similar to this, a recent study has suggested that an imbalance in the RAAS may be involved in COVID-19-pathophysiology [6].

Absolute or relative AT-II deficiency can now be addressed by administering exogenous AT-II. Compared with placebo, this approach reduced 28-day-mortality in patients with a catecholamine-resistent vasodilatory shock and high renin-levels [5]. Based on the available evidence, it can be hypothesized that individualized treatment of patients with vasodilatory shock and elevated renin-levels with exogenous AT-II can improve patient-centered outcomes including survival. Renin measurement is inexpensive and a widely available test.

These considerations may be relevant to cardiac-surgery patients. Kullmar and colleagues [7] showed that a hyperreninemia after cardiac-surgery was associated with a cardiovascular instability and an AKI, but not with an increased mortality. Although Kullmar’s study was affected by limited power because of the low mortality rate, the discrepancy between both studies may also relate to the time points of renin measurements. Kullmar and colleagues measured the renin-levels early after surgery, whereas Flannery and colleagues measured the concentrations once an AKI was present. These observations suggest that elevated renin-levels during the course of a disease might be induced by different causes and may be associated with different outcomes. It is possible, for example, that early increases in renin-levels are primarily caused by reduced ACE-activity, whereas elevated renin-levels seen at a later stage of the disease may be caused by an inadequate activation of the ATR1-receptor. Future research should focus on the nature and causes of AT-II deficiency.

High renin-levels portend a worse outcome and outperform lactate as prognostic indicator of survival [8, 9]. Thus, the notion of a high-renin biotype is becoming increasingly supported by evidence and, given the availability of AT-II, is now clinically relevant [5]. An important additional consideration is the etiology of such elevated renin state. The renin-levels seen in shocked patients exceed levels seen in renin-secreting tumors, which suggests a broad RAAS response. Moreover, since these elevated renin-levels are seen in both severe AKI and ESRD, it is likely that the kidney is not the sole source of renin as this hormone can be secreted from various non-renal sources [5, 10]. This raises the question as to whether patients with high-renin shock have hyperreninemia because of decreased AT-II production or because of decreased ATR1 responsiveness or both or even because of other mechanism [11]. This question cannot yet be answered. However, we know that, in patients with vasodilatory shock, secondary hyperreninemia renin is decreased when AT-II is administered [5]. Additionally, experimental models of sepsis suggest that decreased AT1R-responsiveness may also be important and contribute to kidney injury [12]. Clearly, renewed interest in the RAAS during critical illness demands more sophisticated analyses of all aspects of this system not only at baseline but also over time and in response to interventions.

## Conclusions

There are profound and clinically relevant disturbances of the RAAS in critically ill patients, detected by the presence of hyperreninemia. Depending on the time of renin measurement and the population, this hormone may be associated with the development of AKI or an increased mortality or both. In addition, the vasodilatory biotype of high-renin shock may, in the future, be further defined by the mechanisms responsible for hyperreninemia. Irrespective of the mechanisms, however, personalized treatment of critically ill patients who have high renin-levels with AT-II might improve outcome. The importance and complexity of the RASS in critical illness can no longer be ignored.

## Data Availability

Not applicable.
